# Water footprint in gold extraction: A case-study in Suárez, Cauca, Colombia

**DOI:** 10.1016/j.heliyon.2021.e07949

**Published:** 2021-09-06

**Authors:** Christian E. Alvarez-Pugliese, Fiderman Machuca-Martínez, Mario Pérez-Rincón

**Affiliations:** a Escuela de Ingeniería Química, Universidad del Valle, Cali, Colombia; b Instituto Cinara, Universidad del Valle, Cali, Colombia

**Keywords:** Cyanide, Mercury, Mining, Water use, Water foodprint

## Abstract

This research deepens the analysis of the mineral water footprint, especially that of gold, in regions that are understudied and where mining has been an intensified extractive activity since the colonial era, as is the case in the northern part of department of Cauca in Colombia. Thus, the purpose was to estimate the water footprint indicators in gold mining in Suárez (Cauca, Colombia), to quantify the impacts generated by the non-returned water in the production process and the levels of pollutants in the wastewater, aimed to strength public policies, control strategies and mitigation that generates reductions in the impacts from mining activities on the environment. The blue water footprint was estimated in 79.91 m^3^ per kg of gold extracted and the gray water footprint was found to be in the range of 272,125.39 to 404,825.11 m^3^ per kg of gold extracted. The water footprint values obtained were compared with other mines with similar operations. These results generate a baseline for decision making, providing elements for environmental strategic planning, regulations and showing the great environmental pressure that gold activity exerts on water resources and the territories.

## Highlights

• Assessment of water footprint of gold production in Suárez (Cauca, Colombia).
• Gray water footprint was found to be in the range of 272,125.39 to 404,825.11 m^3^ ​per kg of gold extracted.
• The gold production had much larger gray water footprint than blue water footprint.
• Gold mining activity requires important management by environmental authorities and polluting agents.

## 1 Introduction

Seventy-one percent of the earth's surface is covered by water; however, less than 1% of the planet's potable water is suitable for human consumption [1]. Colombia ranks sixth among the world's countries with the greatest availability of renewable water resources, after Brazil, Russia, Canada, United States and China [2], and first in water availability per km^2^. However, the distribution of water is not homogeneous across the Colombian territory, and the outlook is troubling due to excessive human pressure from population growth and, above all, by the recent specialization of Colombia's economy towards primary sector production, which uses water intensively [3].

In Colombia, the largest water consumers are the agricultural, energy and livestock sectors, which constitute 76.6% of the domestic total water use. According to official records, the mining sector demands 1.8% of total domestic water consumption. Considering that 82% of gold production is alluvial-type exploitation (direct exploitation of the riverbed), of which 95% corresponds to illegal mining [4], and that this type of activity uses pollutant chemicals in its extraction process, the risks of contamination and the generation of wastewater contaminated with mercury and cyanide are high and can have drastic impacts on human populations and ecosystems.

The productive dynamics of the gold mining sector in Colombia increased approximately 6% annually on average from 2000 to 2017, driven by an increase in the international price of gold. In 2017, production totaled 46.2 tons, of which 48.5% was reported to have been mined from areas with mineral titles, 47.1% was reported as production from *barequeo* (panning) and the remaining 4.4% came from special reserve areas and scrap recovery. The production level in 2017 was 34% lower than that in 2016, which may be explained by the implementation of control measures by the Colombian authorities on illegal mining [5, 6].

There does not seem to be any indication that gold will cease to play a key role in the medium- or long-term as an international source of value, nor is there a viable substitute for its role as an essential component in new technological industries, which is why a constant demand persists for this scarce resource [7], implying future increases in pressure on water resources required by this sector. These impacts are reflected in environmental and public health problems around the world [8, 9]. For example, the process of gold extraction is directly responsible for the degradation of ecosystems due to the extraction of vegetation related to mining and soil excavation [10]. The extraction and processing of gold are also important sources of hazardous chemical substances such as mercury, cyanide and arsenic compounds, which have severe impacts on biodiversity and human health [11].

In fact, Colombia's water sources have one of the highest levels of mercury contamination in the world [12]. According to the Atlas of Environmental Justice (www.ejatlas.org), Colombia is also one of the countries with the most reported cases of environmental conflicts, where mining, and especially gold mining, stands out. This situation is explained by the promotion of an extractive model by recent governments, particularly since 2000, accompanied by the increase in international gold prices that have generated a boom and uncontrolled exploitation to the detriment of the environment [13].

In this sense, to quantify the impact generated by human activity on water and in the context of such concepts as ecological footprint, carbon footprint and virtual water flows, the concept of the water footprint (WF) emerges [14, 15], which is a multidimensional indicator that estimates the volume of fresh water used to produce a good or extract a resource (in the case of mining) along the productive or extractive chain; it is primarily used in the study of hydrographic basins or specific geographic areas in a defined time scale, contributing to improved decision making in water management and a determination of the appropriate volume of water for humans [16].

In the "*ENA-National Water Study 2014"* [17], it was estimated that the water demand by the coal and gold mining sector in Colombia was 640.6 Mm^3^/year, with a projection of 948.3 Mm^3^/year by 2022. Unfortunately, this study did not apply the methodology of the water footprint for this sector, with only the blue water footprint indicator being estimated for the petroleum mining subsector, which yielded a value of 6.6 Mm^3^/year [18].

Detailed studies on the analysis of the mineral water footprint are scarce in Colombia. One of the few existing studies was conducted in the mining municipality of Segovia (Antioquia, Colombia), where a gray water footprint indicator was estimated for the gold mining activity in various scenarios, yielding values from 870.45 Mm^3^/year up to 3,650.06 Mm^3^/year [19]. The orders of magnitude of the indicators identify the strong environmental impacts caused by the mining process, in this case gold, on the water sources. Thus, complaints against gold mining in Colombia due to water grabbing and water pollution are often expressed in aphorism that “*water is more valuable than gold*” in non-monetary standards of value [13].

Therefore, it is appropriate to deepen the analysis of the mineral water footprint, especially that of gold, in regions that are understudied and where mining has been an intensified extractive activity since the colonial era, as is the case in the northern part of department of Cauca (Colombia). Thus, the present study estimates the water footprint indicator for gold mining in the municipality of Suárez (Cauca, Colombia), referencing the Water Footprint Assessment Manual [16] and the Guide for the Evaluation of the Water Footprint of Gold and Coal Mining in Colombia [20]. The objective of this work is to contribute to the methodological knowledge for the determination of the water footprint as a measurement, documentation and optimization factor in resource use, aimed at strengthening public policies, control strategies and mitigation that generates reductions in the impacts from mining activities on the environment, especially on water resources. However, a secondary objective, is to provide elements for environmental strategic planning and regulation, which could limit extraction and mining activity in certain regions of the country.

## 2 Methods and data

The article is based on data retrieved from primary sources. It is inevitable that any error in these sources may influence the outcome of this analysis. However, the data has been checked with other sources (Cinara Institute and Colombian Geological Service) and is sufficiently consistent for the analysis.

The information base used for the research was consulted in the Colombian Mining Information System [6], which corresponds to the official source of statistics for the country's mining sector. Likewise, reports from the Colombian Geological Service [21], the Autonomous Regional Corporation of Cauca [22, 23, 24] and other Colombian organizations related to the subject [4, 25], were used.

### 2.1 Description of the studied area

The mining district of El Tambo-Dovio, which includes a large part of the department of Valle del Cauca and part of the north of the department of Cauca, is located in the south-western part of Colombia. In this district, mining products are extracted, such as limestone, construction materials, coal, gold, silver, platinum, among others [26]. Specifically, regarding gold mining, the municipalities of Buenos Aires, Santander de Quilichao and Suarez located in northern Cauca are the ones that represent the highest production in the district. Suárez has a large mining tradition since the colonial era [27], with gold mining activities located in the vicinity of La Salvajina dam (total capacity 908.62 Mm3), the department's largest freshwater reservoir that captures the water from the Cauca river, the second fluvial artery of Colombia (152 m^3^/s average flow rate at La Salvajina dam discharge), with annual precipitations in the zone that varies between 2000 and 3000 mm.

**Figure 1:**
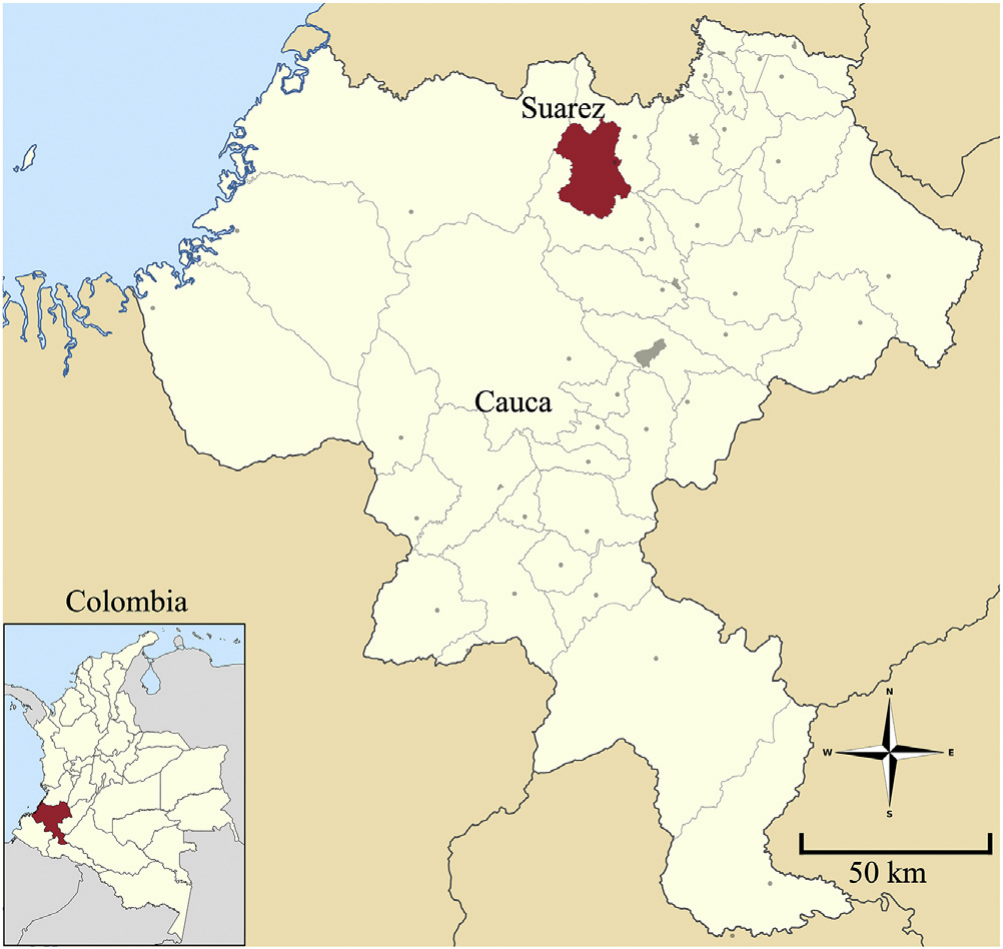
Location of the municipality of Suárez, Cauca (Colombia).

The geographical scope of the study was limited to the area of gold mining in Suárez (Figure 1 showing Suarez in the Cauca department map) covering an area of approximately 390 km^2^ [22]. Geographically it is located on the western mountain range, parallel to the Cauca river, the Ovejas river and the Salvajina dam.

The data reported in the present study are from the mines of the Mindalá and La Toma subdivisions of the municipality of Suárez (Cauca), where the exploitation of gold is mainly done in primary deposits. In the case of alluvial mining, data from the deposits in the Ovejas river, Cauca river, Inguitó river, Quebrada Saladito and Quebrada San Martin [21, 22, 24] were used.

From the geological point of view, the gold in Suárez (Cauca) is in the stock of Pasobobo-Damián which has hydrothermal deposits associated with copper and gold porphyries of dacitic and andesitite composition (Calco-alkaline) emplaced in oceanic affinity basaltic rocks [21]. The alluvial exploitation zones are found in the basins and micro-basins of the Cauca river, Ovejas, Maraveles, La Estrella, Inguito and the San Miguel creek. Geograpichaly the areas where gold is exploited are located in the Matrecaña, Tamboral, Maraveles townships of La Toma corregimiento [23].

**Figure 2:**
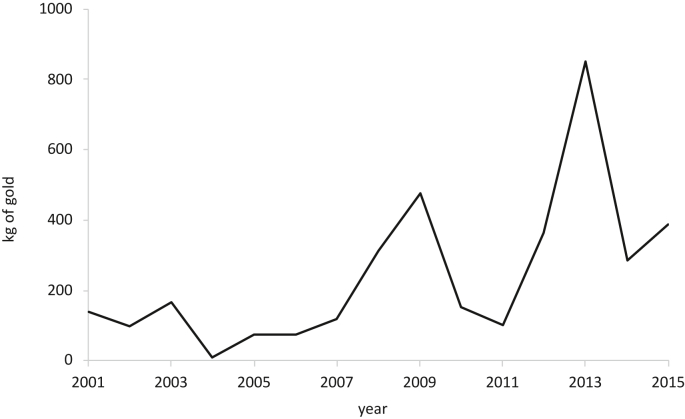
Kilograms of gold per year produced in Suárez 2011–2015 (Cauca, Colombia) (-).

Source: Unidad de Planeación Minero Energética (2018).

In Figure 2, the behavior of the official records of gold production in Suárez from 2001 to 2015 is observed, where a total production of 3.6 tons of gold is reported during the 15 years, with a growth trend that is consistent with the sharp rise in the international price of gold since 2001 to its peak in 2012; the subsequent drop in the international price seems to have contributed to a lower production of the metal in the following years. The fall in production in 2010 can also be explained by the decision taken in 2009 by the company AngloGold Ashanti to abandon all the projects in the area, due in large part to the resistance of the communities.

#### 2.1.1 Mines sample

In the inventory of mineral processing plants, 24 plants or processing sites were identified, of which 19 correspond to plants located in the primary deposits and 6 are sites of alluvial mineral exploitation (Table 1). It should be noted that 12 processing plants were based exclusively on the gravimetric recovery of gold (gravity separation primarily due to weight and size differences between different particles, separated by panning, sluicing or tabling [28]) 4 plants used amalgamation and 4 cyanidation.

**Table 1:** Inventory of the mineral processing plants in Suárez (Cauca, Colombia).

Mine name∗ (location)	Source	Deposit type	Crushing (# of equipments)		Type of beneficiation process (0: Not present 1: Present)			Cyanidation tank volume m^3^
			Hydraulic hammers	Drums	Gravimetric	Amalgamation	Cyanidation	
JL (La Toma)	1,3	Primary	0	1	0	1	0	0
MC (La Toma – El Carmen)	1,3	Primary	4	1	1	0	0	0
FB (La Toma - El Carmen)	1,3	Primary	2	0	1	0	0	0
JF (La Toma – El Carmen)	1,3	Primary	0	8	0	1	0	0
HA (Gelima)	1,3	Primary	5	2	1	0	0	0
TEC IL	1,3	Primary	4	1	1	0	0	0
EA LL	1,3	Primary	3	1	1	0	0	0
JI (El Desquite)	1,3	Primary	0	1	0	1	1	8
PC (El Desquite)	1,3	Primary	3	0	1	0	0	0
PM (El Bosque – Maravelez)	1,3	Primary	3	2	1	0	0	0
WM (Maravelez)	1,3	Primary	3	0	1	0	0	0
HR (Maravelez)	1,3	Primary	0	6	0	1	0	0
AAA (Tamboral)	1,3	Primary	3	0	1	0	1	22
LV (Tamboral)	1,3	Primary	3	0	1	0	0	0
MC FL	1,3	Primary	4	0	1	0	0	0
LVC (El Calvario)	1,3	Primary	4	1	1	0	0	0
AGC (El Danubio)	1,3	Primary	4	1	1	0	1	32
EL (La Carolina)	1,3	Primary	4	1	1	0	0	0
WB	2	Primary	N/A	N/A	1	0	1	167
Quebrada saladito CC	3	Alluvial	0	0	1	1	0	0
Quebrada San Martín	3	Alluvial	0	0	1	1	0	0
Río Cauca (San francisco)	3	Alluvial	0	0	1	1	0	0
Río Ovejas	3	Alluvial	0	0	1	1	0	0
Río Inguitó	3	Alluvial	0	0	1	1	0	0

∗ Due to security reasons names were coded in this research.

Amalgamation with mercury is a process where the concentrated mineral is mixed with mercury to form an amalgam (mercury alloy with gold and silver). Subsequently, the amalgam is burned to evaporate the mercury and recover the gold and silver in its metallic form. This process is highly inefficient because only a fraction of the gold, approximately 30% of the potential to be extracted, is recovered. In small-scale mining, the owners of the independent gold processing sites typically retain the wastes from the amalgamation as payment from the miners for the use of the equipment; these wastes or tailings are subsequently cyanided to extract the remaining gold [29].

The cyanidation process consists in a leaching stage, where the cyanide dissolves the metallic gold in the form of a gold-cyanide complex, thus allowing it to be separated from sand, rocks and other minerals. Subsequently, two techniques may be used to reverse the process in the liquid solution, converting gold from its concentrated gold-cyanide form to metallic gold. The first consists of the precipitation of metallic gold promoted by the addition of zinc, a technique that is widely used in Colombia; the second is the electrodeposition of gold in an electrolytic cell that requires other prior processes of concentration and a higher technological degree, and although this technology is the most widely used worldwide, it is not applied in Colombia [30].

For the exploitation of alluvial ores, it was considered that the extraction was done with mini dredges or backhoes; the crushing and sorting was carried out manually without mechanical aids, and the refinement of the gold was carried out by artisanal techniques (gravimetric concentration) and mercury amalgamation in channels or buckets.

The inventory was provided by the Colombian Geological Services, and the database is considered a secondary source, thus the possibility of measurement, misclassification or selection bias are not excluded. Onsite verification of the mines operation was not possible due to public order problems in the zone. Is recommend for future studies to obtain a remote sensing analysis for small-scale gold mining of the region.

### 2.2 Water footprint

The water footprint (WF) methodology is widely used in the study of hydrographic basins or specific geographic areas in a defined time scale, accounting for water consumption and pollution throughout different anthropogenic activities. The methodology of this study is based on the four phases described in the "The Water Footprint Assessment Manual" [16] and the "Manual for the approach to the evaluation of the water footprint of gold and coal mining in Colombia” from the Energy Mining Planning Unit [20] that serves as a methodological base and provides tools for the analysis of mining activity under the water footprint approach.

The WF of a product is composed of: i) Direct WF (DWF), which refers to water consumed and contaminated in all the production stages and; ii) Indirect HH (IWF), which refers to water consumed and contaminated in the production of goods and services used in the production stages [16]. Where water consumption generates opportunity costs for other social groups and ecosystems that demand the resource [31]. Both, DWF and IWF, are composed of Green Water Footprint (gWF), Blue Water Footprint (BWF) and Gray Water Footprint (GWF), which are explained below. For this work, only DWF was estimated and in particular BWF and GWF are calculated, since gWF (rainwater) is insignificant or null for the extractive gold process.

In this case, the indicators were calculated with the water flows and pollutant quantities estimated from the inventory described in Table 1. The inventory information allowed the construction of a baseline scenario where a production of 328.5 kg of pure gold per year was estimated, a value that is within the official production range in the analysis period (2001–2015), where there is a maximum of 850 kg/year and a minimum of 10 kg/year, with an average of 241 kg/year. This inventory allows to characterize the gold production of the municipality and calculate the BWF and GWF indicators per kg of gold produced.

The calculations and assumptions used for each of the variables studied are explained in the following lines.

#### 2.2.1 Crushing capacity (ton/day)

In the primary ore extraction plants, the number of tons per day of crushed or primary material in a mine was calculated, considering that 1 hydraulic hammer has a crushing capacity of 2.5 ton/day and 1 drum of 0.4 ton/day [25]. With the exception of the JI (El Desquite) mine, where the volume of the cyanidation tank was used to estimate crushing capacity (in cyanidation, 30% is mineral and 70% is water) [22], and the WB mine, where the crushing capacity value is obtained from 2012 report and which is in the range of medium-scale mining.

In the case of alluvial deposits, data from the Corporación Autónoma Regional del Cauca (2006a) [24] were used, where grams of gold extracted per day for each location are reported. The number of tons processed (crushing capacity) was calculated by dividing the grams of gold extracted by 10g/ton, the average grade of gold in alluvial deposits of the zone [24] and the recovery capacity (Table 2). The values of each plant were added, and the annual value was calculated in tons/year. A total of 244 business days per year were considered for the annual calculations.

**Table 2:** Gold recovery capacity by type of beneficiation.

	Gravimetric^1^	Amalgamation^2^	Cyanidation^3^	Gravimetric + Amalgamation	Amalgamation + Cyanidation	Gravimetric + Cyanidation
Recovery Capacity (percentage of recovery, %)	45.0%	30.0%	89.5%	61.5%	92.7%	94.2%

#### 2.2.2 Gold production Law 750 (g/day)

The amount of gold extracted with a purity of 75% (average quality produced by small and medium-sized mining operations) was calculated by multiplying the crushing capacity of each mine by the average grade, which for alluvial deposits is 10 g/ton and for primary deposits is 15 g/ton [24]. The resulting value was multiplied by the average recovery capacity of each type of beneficiation (Table 2). In the case of mines with more than one type of beneficiation process, the percentage of accumulated recovery was calculated, considering the average local recovery of each stage (Table 2).

In the case of alluvial deposits, data from the Corporación Autónoma Regional del Cauca (2006a) were used, where the grams of gold extracted per day for each location are reported.

#### 2.2.3 Gold production Law 999 (g/day)

The amount of gold reported in the official records of the Mining and Energy Information System [6] corresponds to the pure gold Law 999 (99.9% purity). To convert Law 750 to Law 999, the amount of Law 750 gold is multiplied by the conversion factor 0.75.

#### 2.2.4 Water inflow (m^3^/day)

Water inflow at the mine was estimated by multiplying the specific water consumption required by each type of beneficiation process (Table 3) by the tons crushed in each mine. For plants with more than one type of beneficiation process, consumption is added in each stage because they are independent and fresh water is required in each phase; for example, the outlet water of the gravimetric concentration cannot be reused directly in the cyanidation stage due to its high concentration of solids.

**Table 3:** Specific water consumption by type of beneficiation process.

	Gravimetric^1^	Amalgamation^2^	Cyanidation^3^
Specific water consumption m^3^/ton	6.0	0.3	2.3

#### 2.2.5 Water outflow (m^3^/day)

The water outflow or discharge rate was calculated considering that for the different types of beneficiation process, most of the processing water is returned to the natural water source from which it is collected, and there is a percentage of water that does not return, remaining contained in the moisture of the sludges and sands processed in the primary ore mines. These sands are accumulated in piles with a water content between 9 and 17% [22, 24, 29]. For calculating the water retained in the productive process, the maximum value of 17% was used. Other evaporative processes were not considered during the extractive process because they are deemed negligible.

For the alluvial mines, where the processed sands and sludge are discharged back into the river, the discharge rate is equal to the inflow rate.

#### 2.2.6 Mercury (Hg) used

In the amalgamation process of primary ore mining, an average of 27.5 g of mercury (chemical formula: Hg) per gram of recovered gold are used [25].

For the alluvial deposits, the reported use value of 20.0 g of mercury per gram of gold recovered was used, using the backhoe extraction method and beneficiation by amalgamation in open-flow gutters [25].

#### 2.2.7 Hg discharge method 1

Method 1 considered the study by Ruíz Solano (2016), where the discharges were characterized and a material balance for mercury was performed in a small-scale primary ore mining plant in Cauca, where gold is extracted through an amalgamation process in a semi-closed cycle. In this study, a discharge was estimated in the liquid effluent of 3.38% of the mercury used in each cycle. Likewise, there are losses due to volatilization of 1.64% of the initial mercury in each cycle, which evaporates during the burning of the amalgam and constitutes a source of air pollution (the loss was not included in the discharge calculation because only liquid discharges are considered).

For the alluvial deposits, the reported value of 0.31 g of mercury released to the soil, atmosphere or water per gram of gold recovered in alluvial mines in the semi-closed cycle for the mining area of the municipalities of Buenos Aires and Suárez was used [25]. This value was corrected by multiplying it by a factor of 66%, considering that of the total mercury emissions, approximately 66% corresponds to the discharge into the liquid effluent [32].

#### 2.2.8 Hg discharge method 2

For the primary deposits, the reported value of 7.7 g of mercury released to the soil, atmosphere or water per gram of gold recovered in the open cycle for the mining area of Buenos Aires and Suárez was used [25].

For the alluvial deposits, the reported value of 15.5 g of mercury released to the soil, atmosphere or water per gram of gold recovered in alluvial mines in the open cycle for the mining area of Suárez was used [25].

These values were corrected with a factor of 66%, considering that of the total mercury emissions, approximately 66% corresponds to the discharge in the liquid effluent [32].

#### 2.2.9 Cyanide (CN) used

According to the Mining-Environmental Assessment of the Suárez Mining District [22], in the cyanidation process of primary ore mining, an average of 1.5–6.0 kg of sodium cyanide per m^3^ of solution is used. To calculate the use of CN, an average value of 3.75 kg of sodium cyanide per m^3^ of solution was used. This value was multiplied by the volume (in m^3^) of the cyanidation tanks of each plant.

#### 2.2.10 CN discharge

A cyanide analysis performed by the Colombian Geological Service on cyanide-poor solutions at three mineral processing plants in Buenos Aires and Suárez showed that on average, this effluent had a concentration of free cyanide of 1.5 g/l [21]. This value was established as the concentration of the discharge effluent, signifying that 40% of the cyanide is expelled from the mine in the liquid effluent.

#### 2.2.11 Blue water footprint (BWF)

BWF is an indicator of the consumptive use of water, which corresponds to fresh water extracted from a surface water body or from groundwater for the development of a productive or extractive activity [16]. In this case, the BWF is the amount of water consumed by the mining process. This calculation was based on the balance of the inputs and outputs of water from the mine. Considering that the difference in the volume of water entering and exiting the mine corresponds to the evaporation and/or incorporation of water in the product. Eqs. (1) and (2) present the calculated indicators.

(1) $$BW=Water{\;}_{inflow}-Water{\;}_{outflow}$$

(2) $$BWF\;Indicator=\frac{BWF}{Production}$$

where $\;Water_{iniflow}$ is the total water input to the process (aqueduct, underground, surface, rain) in m^3^/year; $\;Water{\;}_{outflow}$ is the sum of the total water discharge in the process in m^3^/year; and Production is the kg of gold per year produced per unit of analysis (zone, mine, municipality, etc.).

#### 2.2.12 Gray water footprint (GWF)

GWF is an indicator of the degree of contamination of fresh water associated with the productive or extractive process. It is defined as the volume of fresh water (from an uncontaminated source) required to dilute the pollutant loads produced by the production process until the concentration of water contaminants reaches the natural conditions of the source and the existing water quality standards [16]. In this case, the GWF represents the volume necessary to dilute a contaminant present in the discharges up to a value that guarantees the quality of the water in the receiving water body. The indicator is calculated according to Eqs. (3) and (4).

(3) $$GW=\frac{Flow_{out}*\;Concentration_{out}-Flow_{in}*\;Concentration_{in}}{Concentration_{max}-\;Concentration_{nat}}$$

(4) $$GWF\;Indicator=\frac{GWF}{Production}$$

where $\;Flow_{out}$ is the outflow or discharge rate of the mine in m^3^/year; $\;Concentration_{out}$ is the concentration of a parameter exiting the mine in g/m^3^; $\;Flow_{in}$ is the inflow to the mine in m^3^/year; $\;Concentration_{in}$ is the concentration of a parameter entering the mine in g/m^3^; $\;Concentration_{max}$ is the maximum permissible concentration of the contaminant to maintain the quality of the receiving effluent of the discharge in good condition in g/m^3^; $\;Concentration_{nat}$ is the natural concentration of the contaminant (without anthropogenic intervention) in g/m^3^; and Production is the kg of gold per year produced per unit of analysis (zone, mine, municipality, etc.).

In the case of pollutants such as cyanide or mercury, which do not come from the water entering the mine as all discharge is due to the activity performed inside the mine, the GWF may be calculated according to the following modification of Eq. (3):

(5) $$GWF=\frac{Discharge}{Concentration_{max}}$$

where Discharge is the amount of pollutant discharged in the mine's effluent in g/year.

The maximum allowable concentration of CN discharge is 1 mg/l, and Hg is 0.002 mg/l for gold extraction activity, according to Resolution 0631 of the Ministry of the Environment and Sustainable Development.

For the calculation of the GWF of each processing plant or site of exploitation, it was considered that the CN and the Hg discharges were performed in independent effluents even if a mine had both cyanidation and amalgamation processes.

Note that one of limitations of the water footprint methodology applied to highly polluting activities such as mining can lead to gray footprint calculation to be infinite due to strict maximum allowable discharge concentration of pollutants.

## 3 Results and discussion

Based on the mine sample from Table 1, the baseline scenario with the estimated values of the different consumptions and indicators is presented in Table 4. It is important to highlight the percentage of gold production in the municipality according to the type of deposit estimated for the baseline scenario, which was 97.7% for primary ore mining and 2.3% for alluvial mining, values that agree with previous reports [22, 24, 27] that mention underground gold mining is the dominant type of production in the Suárez. A 2016 study [4] used satellite images and other remote sensing tools to quantify the number of hectares affected by mining in Colombia, determining that the department of Cauca has the least impact among the seven gold producing departments, according to the authors this may be due to a lack of accountability for primary ore mining in the remote sensing method used. Recent remote sensing studies in Africa and South America have successfully account for the footprint of small-scale artisanal gold mining [33, 34].

**Table 4:** Baseline scenario for the estimation of the WF of gold mining in Suárez (Cauca, Colombia).

Mine name and/or location	Crushing capacity ton/day	Gold production Law 750 g/day	Gold production Law 999 g/day	Water in m^3^/day	Water out m^3^/day	BWF m^3^/day	Hg used g/day	Hg discharge method 1 g/day	Hg discharge method 2 g/day	GWF CN used kg/day	CN discharge kg/day	TOTAL GWF Method 1 m^3^/day	TOTAL GWF Method 2 m^3^/day
JL (La Toma)	0.4	1.8	1.4	0.1	0.1	0.0	49.5	1.7	9.1	0.0	0.0	838.4	4573.8
MC (La Toma – El Carmen)	10.4	70.2	52.7	62.4	56.9	5.5	0.0	0.0	0.0	0.0	0.0	0.0	0.0
FB (La Toma - El Carmen)	5.0	33.8	25.3	30.0	27.4	2.6	0.0	0.0	0.0	0.0	0.0	0.0	0.0
JF (La Toma – El Carmen)	3.2	14.4	10.8	1.0	0.9	0.1	396.0	13.4	73.2	0.0	0.0	6707.2	36590.4
HA (Gelima)	13.3	89.8	67.3	79.8	72.8	7.0	0.0	0.0	0.0	0.0	0.0	0.0	0.0
TEC IL	10.4	70.2	52.7	62.4	56.9	5.5	0.0	0.0	0.0	0.0	0.0	0.0	0.0
EA LL	7.9	53.3	40.0	47.4	43.2	4.2	0.0	0.0	0.0	0.0	0.0	0.0	0.0
JI (El Desquite)	2.4	33.4	25.0	6.4	5.8	0.6	297	10.1	54.9	30.0	12.0	17030.4	39442.8
PC (El Desquite)	7.5	50.6	38.0	45.0	41.0	4.0	0.0	0.0	0.0	0.0	0.0	0.0	0.0
PM (El Bosque – Maravelez)	8.3	56.0	42.0	49.8	45.4	4.4	0.0	0.0	0.0	0.0	0.0	0.0	0.0
WM (Maravelez)	7.5	50.6	38.0	45.0	41.0	4.0	0.0	0.0	0.0	0.0	0.0	0.0	0.0
HR (Maravelez)	2.4	10.8	8.1	0.8	0.7	0.1	297	10.1	54.9	0.0	0.0	5030.4	27442.8
AAA (Tamboral)	7.5	106.0	79.5	62.5	57.0	5.5	0.0	0.0	0.0	80.6	32.3	32250.0	32250.0
LV (Tamboral)	7.5	50.6	38.0	45.0	41.0	4.0	0.0	0.0	0.0	0.0	0.0	0.0	0.0
MC FL	10.0	67.5	50.6	60.0	54.7	5.3	0.0	0.0	0.0	0.0	0.0	0.0	0.0
LVC (El Calvario)	10.4	70.2	52.7	62.4	56.9	5.5	0.0	0.0	0.0	0.0	0.0	0.0	0.0
AGC (El Danubio)	10.4	147.0	110.2	86.7	79.1	7.6	0.0	0.0	0.0	120.0	48.0	48000.0	48000.0
EL (La Carolina)	10.4	70.2	52.7	62.4	56.9	5.5	0.0	0.0	0.0	0.0	0.0	0.0	0.0
WB	50.0	706.7	530.0	416.7	380.1	36.6	0.0	0.0	0.0	625.0	250.0	250000.0	250000.0
Quebrada saladito CC	2.2	10.0	7.5	14.0	14.0	0.0	200.0	3.1	50.8	0.0	0.0	1550.0	25410.0
Quebrada San Martín	2.2	10.0	7.5	14.0	14.0	0.0	200.0	3.1	50.8	0.0	0.0	1550.0	25410.0
Río Cauca (San francisco)	1.6	7.0	5.3	9.8	9.8	0.0	140.0	2.2	35.6	0.0	0.0	1085.0	17787.0
Río Ovejas	2.2	10.0	7.5	14.0	14.0	0.0	200.0	3.1	50.8	0.0	0.0	1550.0	25410.0
Río Inguitó	1.1	5.0	3.8	7.0	7.0	0.0	100.0	1.6	25.4	0.0	0.0	775.0	12705.0
**TOTAL (per day)**	194.2	1795.1	1346.3	1284.6	1177.0	107.6	1879.5	48.2	405.5	855.6	342.3	366366.3	545021.8
	**ton/year**	**kg/year**	**kg/year**	**m**^**3**^**/year**	**m**^**3**^**/year**	**m**^**3**^**/year**	**kg/year**	**kg/year**	**kg/year**	**kg/year**	**kg/year**	**m**^**3**^**/year**	**m**^**3**^**/year**
	47,392.9	438.0	328.5	313,444.0	287,192.3	2,6251.8	458.6	11.8	99.0	208,772.5	83,509.0	8,939,3371.5	13,298,5319.2
						**m**^**3**^**/kg of gold**		**m**^**3**^**/kg of gold**	**m**^**3**^**/kg of gold**		**m**^**3**^**/kg of gold**	**m**^**3**^**/kg of gold**	**m**^**3**^**/kg of gold**
						79.9		17912.8	150612.5		254,212.6	272,125.4	404,825.1
								**m**^**3**^**/year**	**m**^**3**^**/year**		**m3/year**		
								5,884,371.5	4,9476,319.2		83,509,000.0		

### 3.1 BWF indicator

The BWF indicator was estimated at 79.9 m^3^/kg of gold, which is higher than the values reported in Segovia (Antioquia) – 58.69 m^3^/kg of gold [19] – and in the "Reina de Oro" mine in Vetas (Santander) – 21.79 m^3^/kg of gold [35]. Both mines use similar mineral beneficiation processes typical of small- and medium-scale mining operations. However, the value was low when compared to the BWF estimated in the study by Haggard (2015), where 228 m^3^/kg of metal production was calculated for a plant in South Africa with large-scale mining operations that extracted precious metals from the platinum group (ruthenium, rhodium, palladium, osmium, iridium and platinum). For the BWF indicator, there is a greater incorporation of water in the production processes in large-scale mining operations compared to small- and medium-scale mining operations due to modernization, which includes a greater number of refining stages and auxiliary services, such as refrigeration, cleaning and hydraulic transport, among others, that increase the water consumption per kg of metal produced.

Table 5 presents the values for the estimated annual BWF from 2001 to 2015, considering the official gold production reported at Suárez. These values are between 838.1 and 67,940.2 m^3^/year; however, these estimations are only comparable between mines or mining sectors with an equal or similar capacity of gold production and with similar extraction technologies; due to the limited number of studies on the subject, these results could not be extrapolated at the national level.

**Table 5:** BWF of gold mining in the period from 2001 to 2015 in Suárez (Cauca, Colombia).

	2001	2002	2003	2004	2005	2006	2007	2008	2009	2010	2011	2012	2013	2014	2015
BWF m^3^/year	11006.4	7745.7	13375.8	838.1	6021.9	5810.6	9454.4	25058.9	38076.1	12290.4	7989.1	29092.1	67940.2	22678.5	31078.4

Given the lack of estimates of water footprint indicators at municipality level in the department of Cauca, we proceeded to compare measurements with values of BWFs reported for other sectors in other municipalities in Colombia. For example, the evaluation of the Water Footprint in the Porce river basin [36] reported for the municipality of Gómez Plata (Antioquia) which has an area (360 km^2^) and a population (16,101 inhabitants) similar to the municipality of Suárez (390 km^2^ and 18,656 inhabitants), a BWF in the domestic sector of 40 m^3^ per inhabitant per year is equivalent to a total of 644,040 m^3^/year. The BWFs of other sectors in Colombia, such as the hydroelectric (24 Mm^3^ per year), livestock (12 Mm^3^/year) and agricultural (14 Mm^3^/year) sectors, are reported for the total Porce river basin. A study by Pérez et al. (2017), which estimated the BWF for fish farming in Colombia, reported the following BWF for three types of fish farms in the department of Valle del Cauca: 2.0 m^3^/kg for tilapia, 0.8 m^3^/kg for Blackfin Pacu and 0.1 per kg for trout. From the analysis of the different BWFs, it can be inferred that gold mining, compared to some of the cases observed in other sectors, is an activity that retains a relatively high quantity of blue water. Thus, the extraction of 1 kg of gold retains two times more water than the domestic use of one person in a year, and between 4 to 14 times the amount of water required to obtain 1 kg of farmed fish.

### 3.2 GWF indicator

For the base case GWF indicator (Table 4), two methods were used (based in the two methods for Hg explained in section 2.2). A value of 272,125.4 m^3^/kg of gold was estimated for method 1 and 404,825.1 m^3^/kg of gold with method 2. In method 1, the amalgamation stage is performed in a semi-closed process with a higher percentage of recycled mercury; therefore, mercury contamination (Hg) represents only 7% of the GWF and the balance is due to cyanide (CN). In method 2, mercury is not it is not reused, thud 37% of the GWF corresponds to mercury contamination.

The amalgamation process is prohibited in Colombia due to the toxic and bioaccumulable nature of this heavy metal, which damages the brain, kidneys and testes, causes neurodevelopmental and behavioral delays, subtle damage to visual memory, attention and speed deficits in visual responses, attention deficit, hearing and psychomotor damage, severe skin inflammation, irritation of the gastrointestinal tract, severe hepatic damage and progressive and generalized paralysis of the extremities, among other problems [37]. The Colombian law 1658 of 2013 and the Minamata Agreement in March 2018 represent Colombia's commitment to the scheduled phase-out of mercury use in mining by 2018 and in various industrial activities by 2023. Nevertheless, the severity of mercury contamination in Colombia, as previously stated, ranks as one of the highest levels globally [12]. In a study that measured the mercury content on water samples from Cauca river in Suárez from 2018 and 2019 found mercury from the operations in the amalgamation process in concentrations up to 0.010 mg/l, five higher the maximum discharge limit [38].

The contamination by cyanide contributes the most to the calculation of the GWF because the cyanidation technique is used in the Suárez plants with greater crushing capacity. It is important to note that cyanide contamination has a short-term effect and is not bioaccumulable (with the exception of some complexes) because it can be partially degraded by the sun's ultraviolet radiation [39]; however, the possible formation of soluble complexes of cyanide with other metals, such as mercury, and the possible breaking of storage dams and tailings spills to water sources can generate the production of hydrocyanic acid (when the water pH reaches values below 11), constituting a serious risk to the environment and human health because cyanide inhibits the transfer of oxygen to cells causing suffocation and death [40].

In general, tailings from the gold industry may be classified into the following types of waters: (1) waters with mercury due to hauling of amalgam, elemental mercury and other chemical compounds formed with mercury during processing; (2) residual cyanide waters from the process of precipitation with zinc and acid waters; and (3) products from the dissolution of metals and other compounds washed away by rainwater in excavation piles and debris. In most cases, this last type of water is mixed during the extraction process. In the present study, the first two types were calculated.

The values estimated in this study are lower than those of the study by the CTA (2013), which calculated the GWF of gold mining to be 1.5 Mm^3^ of water per kg of gold extracted for the processes of amalgamation and cyanidation in small- and medium-scale mining operations in Segovia, Amalfi, Anorí, Remedios and Zaragoza (Antioquia). This difference is explained by the fact that in Suárez, 50% of the processing plants base their production exclusively on gravimetric methods without using toxic chemicals such as cyanide or mercury, whereas for the group of municipalities in Antioquia, the estimations were performed with the use of mercury in all gold extraction plants, which helps explain the fact that municipalities such as Segovia have the greatest number of cases of mercury poisoning in Colombia [41].

In contrast, when comparing the GWF reported for a large-scale mining plant in South Africa (of platinum) [42] the GWF is lower, reporting a value of 578 m^3^/kg of extracted metal. Thus, it is important to highlight that the legal nature of large-scale mining implies the enforcement of environmental regulations is more rigorous and greater controls are placed on the discharge of effluents.

However, in large-scale mining operations, the quantities of extracted metal are measured in tons per year, in contrast to kg per year for small- and medium-scale mining operations; this results in a large impact when calculating the annual GWF (m^3^/year), resulting in values on the order of tens of Mm^3^/year. This does not consider the impact generated after the closure of a mine and the subsequent management required for the tailings or wastes of the mining process, which contain a high concentration of toxic substances that are not included in the GWF calculations of the large-mining sector because they are discharged and stored inside the mine and not discharged to external bodies of water. This is known in legal-environmental language as environmental liabilities.

**Figure 3:**
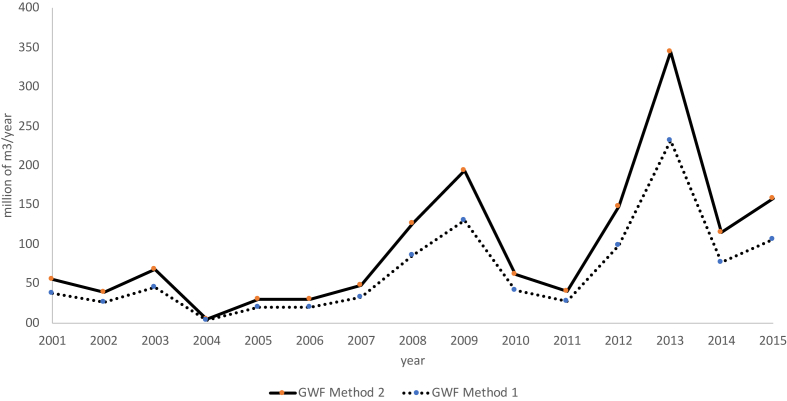
GWF method 1 and method 2, 2001–2015.

Table 6 and Figure 3 present the estimated values of the GWF from the two methods for the years 2001–2015 in the municipality of Suárez, with values between 2.9 and 231.4 Mm^3^/year for methodology 1 and in a range from 4.2 to 344.2 Mm^3^/year for methodology 2. These values are lower than those estimated in the analysis performed for the municipality of Segovia (Antioquia), which yielded GWF values between 870.45 Mm^3^/year and 3,650.06 Mm^3^/year. However, gold production in this municipality was in the range of 1.7–5.5 tons for the years of the study, which represents an order of magnitude higher than the gold production in Suárez.

**Table 6:** GWF of gold mining in the period from 2001 to 2015 in Suárez (Cauca, Colombia).

	2001	2002	2003	2004	2005	2006	2007	2008	2009	2010	2011	2012	2013	2014	2015
GWF Method 1 Mm^3^/year	37.5	26.4	45.5	2.9	20.5	19.8	32.2	85.3	129.7	41.9	27.2	99.1	231.4	77.2	105.8
GWF Method 2 Mm^3^/year	55.8	39.2	67.8	4.2	30.5	29.4	47.9	126.9	192.9	62.3	40.5	147.4	344.2	114.9	157.4

The annualized GWF values in Suárez, using both methodologies, represent a critical environmental situation due to the large quantity of clean water (on average 50 m^3^/s [43], equivalent to 1,576.8 Mm^3^/year) required to dilute contaminated gold production water to a comparative quality of the water flowing in the Cauca river; in other words, for the highest values estimated in methodologies 1 and 2, 15% and 22% of the total river flow rate, respectively, is required to dilute the pollutants to safe levels (with the values of the Pan de Azucar hydrological station located 25 km from Suárez's urban center). This constitute a red flag alert for cities like Cali (2.5 millions of inhabitants) and other municipalities (approximately 10 millions of inhabitants) downstream Suárez, whose drinking water source is mainly the Cauca river, where mines discharge their tailings.

The annual GWF of gold mining in the municipality of Suárez can be compared with GWF estimations in other sectors; for example, in the Porce river basin, which has an area of 5,248 km^2^ and includes 29 municipalities of the department of Antioquia, the GWF of the agricultural sector was estimated in 5 Mm^3^/year and 220 Mm^3^/year for the livestock sector is [36]. These values are significantly lower than GWF of gold mining in the municipality of Suárez, even though the Porce river basin geographic area is 14 times bigger than Suárez and the agricultural/livestock activities occupy large tracts of land and intensively use more direct water. Comparing the intensity of impact per hectare in both areas, sheds light on the high environmental pressure of gold mining. While livestock and agricultural activities added in Porce generate 428 m3/ha of environmental pressure in terms of gray water footprint, the intensity of gold mining in Suárez is between five and ten times that amount depending on the methodology used (2,712 and 4,035 m3/ha).

When the indicators calculated in the present study were extrapolated for the Colombian official total gold production (47.6 ton in 2020) a BWF of 303.9 Mm^3^/year was estimated, while the GWF was found to be between 12,953.1 and 19,269.7 Mm^3^/year. These data prove the great pressures exerted on water resources, both in terms of quality and quantity, from gold mining activities in Suárez and Colombia. Environmental concerns have been accentuating for Colombia and the rest of Latin America due to the accelerated process of economic re-specialization towards the primary sector since the end of the last century, where gold mining is important. In the Colombian case, this concern is accentuated by the decrease in resources for the environmental sector and the participation of violent illegal agents in gold-bearing activities as a way of financing their armed struggle. Both aspects limit the institutional possibilities for greater and better environmental management and control.

### 3.3 Proposals

From the results obtained, strategies and proposals for the gold mining sector were formulated, which may be applied in areas influenced by small- and medium-scale mining operations, such as Suárez. The proposed strategies and recommendations are as follows:

• Implement strategic environmental planning throughout the country to limit and, in many cases, prohibit gold mining activities in territories and areas that are highly fragile, of ecosystemic importance and culturally diverse as well as areas that provide water for human consumption such as tropical dryland forests, mangroves, wetlands and, in general, ecosystems and strategic territories for the conservation of biodiversity, and health of the population and that provide environmental and cultural services.
• Implement the mercury elimination plan in Colombia. Effectively controlling sources of illegal importation of mercury into the country.
• Take advantage of new trends in consumer awareness for cleaner processes that result in lower environmental impacts but acknowledging that "green gold" is not feasible in any of the cases.
• Support technological conversion processes through mining training centers in those populations involved in this economic activity. Facilitate the transformation of medium-scale mining, from amalgamation plants to cyanidation plants, with adequate tailings management, thus achieving reductions in GWF.
• Encourage unions and associations between small-scale miners by developing models that support coexistence between organized groups of small-scale miners and medium- and large-scale mining stakeholders and allowing productive synergies such as the processing of material collected by small-scale miners in medium- and large-scale industrial plants.
• Carefully implement gravimetric concentration processes, which, although they do not use toxic inputs, can lead to an increase in suspended solids in water that, upon reaching rivers or other bodies of water, can decrease the availability of food for fish and reduce the absorption capacity of light in riverbeds, generating imbalances in aquatic ecosystems [4]. Future studies should measure the GWF associated with suspended solids discharged in gold mining processes.
• Promote a green tax on gold extraction that applies to exports or the extractive activity itself. This should be aimed at financing technological conversion, promote research in this field and the construction of wastewater treatment plants.

## 4 Conclusions

In this study, an approximation was made to calculate indicators of the water footprint of gold mining in the municipality of Suárez (Cauca, Colombia), determining that the BWF is 79.91 m^3^/kg of gold extracted and the GWF ranges between 272,125.39 and 404,825.11 m^3^/kg of gold extracted. These values confirm the high impact of gold mining on water bodies caused predominantly by contamination from cyanide (93–63%) and mercury (7–37%), whereas the incorporation of water in the mining process (BWF) is moderately high compared to other productive sectors.

Measurements of the water footprint of gold mining in this small population of Colombia enlighten an analysis of the environmental impacts and true economic importance of this precious metal, in which a process for replacing the current demand for gold in all its markets must be identified, either as a sumptuary good, store of value or raw material for new technologies. Only a paradigm shift will permit societies to assign greater importance to the conservation of environmental heritage over uncontrolled exploitation by the mining industry. In the process of such changes, this analysis allows strategies and proposals to be formulated for the gold mining sector, among which are the implementation of strategic environmental planning throughout the country gold mines and advancing the technological conversion for total elimination of the use of mercury.

## Declarations

### Author contribution statement

Christian E. Alvarez-Pugliese: Conceived and designed the experiments; Performed the experiments; Analyzed and interpreted the data; Contributed reagents, materials, analysis tools or data; Wrote the paper.

Fiderman Machuca-Martínez: Analyzed and interpreted the data; Contributed reagents, materials, analysis tools or data; Wrote the paper.

Mario Pérez-Rincón: Conceived and designed the experiments; Analyzed and interpreted the data; Contributed reagents, materials, analysis tools or data; Wrote the paper.

### Funding statement

This work was supported by Minciencias (669/2014).

### Data availability statement

Data included in article/supplementary material/referenced in article.

### Declaration of interests statement

The authors declare no conflict of interest.

### Additional information

No additional information is available for this paper.
